# Harnessing the power of data linkage to enrich the cancer research ecosystem in Canada.

**DOI:** 10.23889/ijpds.v7i3.1950

**Published:** 2022-08-25

**Authors:** Robin Urquhart, Philip Awadalla, Parveen Bhatti, Trevor Dummer, Simon Gravel, Jennifer Vena, Riaz Alvi, Philippe Broet, Cynthia Kendell, Victoria Kirsh, Guillaume Lettre, Kimberly Skead, Grace Shen-Tu, Ellen Sweeney, Donna Turner

**Affiliations:** 1 Dalhousie University; 2 Ontario Institute for Cancer Research; 3 BC Cancer; 4 University of British Columbia; 5 McGill University; 6 Alberta Heatlh Services; 7 Saskatchewan Cancer Agency; 8 Université de Montreal; 9 Nova Scotia Health; 10 CancerCare Manitoba

## Objectives

We will enrich the cancer research ecosystem in Canada through linking cancer registry and administrative health data to the Canadian Partnership for Tomorrow’s Health (CanPath) cohort and biobank. CanPath is Canada’s largest population health study, including 1% of the Canadian population, which seeks to investigate cancer development.

## Approach

We are achieving record-level linkage of the CanPath harmonized dataset to provincial cancer registry data, and hospitalization and ambulatory care data from the Canadian Institutes of Health Information (CIHI). The CanPATH harmonized dataset includes comprehensive genetics, environment, lifestyle, and behaviour data. Our linkage activities will result in interprovincial data sharing, with centrally-held linked data, a first in Canadian history. We will demonstrate the CanPath-cancer registry-CIHI linkage potential by investigating the impact of the COVID-19 pandemic on healthcare utilization and outcomes among those with cancer.

## Results

The linkage is ongoing and anticipated to be completed by September 2022. Linked data will be made available through the CanPath Data Safe Haven, a cloud-based solution that meets the legal requirements of the data sharing agreements and provincial privacy policies, and is accessible to researchers through secure access. The CanPath Data Safe Haven will be a federated data platform for Canadian researchers to access, analyze, and contribute research in a collaborative environment. By linking these datasets, this project will: address concerns related to accessibility of cancer data in Canada; bring more value to existing data; support an enhanced understanding of the impacts of cancer on marginalized populations; and create a more integrated approach to cancer data access and management.

## Conclusion

CanPath will be the first program in Canadian history to combine the wealth of cohort resources with cancer registry and administrative health data in a central location at a national scale. We will provide a single point of access for researchers to conduct novel investigations into cancer development and outcomes.

